# Transcriptome profiling of human dermal MDPL fibroblasts reveals a characteristic molecular signature providing insights into pathogenic mechanisms

**DOI:** 10.1007/s00109-025-02597-y

**Published:** 2025-10-13

**Authors:** Michela Murdocca, Gerardo Pepe, Serena Maccaroni, Paola Spitalieri, Manuela Helmer-Citterich, Giuseppe Novelli, Federica Sangiuolo

**Affiliations:** 1 Department of Biomedicine and Prevention, University of Rome “Tor Vergata”, Via Montpellier 1, 00133 Rome, Italy; 2 Department of Biology, University of Rome “Tor Vergata”, Via Della Ricerca Scientifica 1, 00133 Rome, Italy

**Keywords:** POLD1, MDPL syndrome, Aging, RNA sequencing, DNA repair

## Abstract

**Abstract:**

The emerging perception that the mammalian dermis encloses fibroblasts with differing functional identities has profound implications for understanding a wide range of genetic pathological states, including aging. MDPL syndrome (mandibular hypoplasia, deafness, progeroid characteristics, and lipodystrophy; MIM #615381) is an extremely rare, genetic progeroid disorder. Patients reported variants in the *POLD1* gene (NM_002691.3), encoding for the evolutionarily conserved catalytic subunit of DNA polymerase delta (Polδ). The protein is a critical enzyme reliable for synthesizing nascent DNA strands in the eukaryotic genome. Importantly, Polδ also serves to repair DNA lesions due to mutagen exposure. As the natural history of MDPL still remains poorly known, we have performed RNA sequencing analyses on human dermal fibroblasts (HDFs) of two MDPL patients, heterozygotes for p.Ser605del, compared to WT HDFs. The bioinformatic analyses identify differentially expressed transcripts related to the extracellular matrix of connective tissue and transduction signal markers. Successively, we shed light on the capacity of MDPL cells to respond to and repair DNA damage by comparing transcript levels between X-irradiated MDPL HDFs and non-irradiated ones. Importantly, the results allowed us to identify specific downregulated molecular traits in irradiated MDPL HDFs, including those genes closely involved in the mechanisms of DNA replication and repair. These data were further validated at the functional level, choosing four pivotal proteins (CDC6 (*Cell Division Cycle 6*), CLSPN (Claspin), XRCC3 (*X-Ray Repair Cross Complementing 3*), RAD51 (*DNA repair protein RAD51 homolog 1*)) involved in interconnected pathways ensuring genomic stability. This work provides critical insights into the pathogenesis and the regulatory mechanisms of MDPL syndrome and related diseases, paving the way for future therapeutic interventions.

**Key messages:**

- We identified a molecular signature in MDPL human dermal fibroblasts by transcriptomic profiling.
- We identified specific markers linked to the extracellular matrix of connective tissue and transduction signal markers.
- We ascertained in irradiated MDPL human dermal fibroblasts specific downregulated molecular traits, involved in the mechanisms of DNA replication and repair.
- We validated at functional and biochemical level specific those proteins involved in pathways ensuring genomic stability.
- The markers identified could be targeted for therapeutic intervention in MDPL syndrome and aging-related diseases.

**Supplementary Information:**

The online version contains supplementary material available at 10.1007/s00109-025-02597-y.

## Introduction

Recent studies exploring the molecular and biochemical features of genetic disorders have emphasized the significant impact of recognizing that mammalian tissues—like the dermis—contain fibroblasts with distinct functional roles, a concept that is reshaping our understanding of many pathological conditions [1, 2].

HDFs are one of the cell types of choice for studying the pathogenic mechanisms underlying aging and premature aging diseases [3], and they have been recently used as target cells for innovative mRNA-based treatments to attenuate the effects of skin aging [4].

The MDPL syndrome (MIM #615381) is an extremely rare progeroid autosomal dominant disorder with few cases diagnosed in the world (approximately 30), characterized by lipodystrophy and mandibular hypoplasia, deafness, telangiectasia, and skin scleroderma. At birth, affected individuals typically exhibit normal weight, size, and appearance, with diagnosis often occurring around 20 years of age. MDPL patients commonly show an unusual life expectancy, compared with other progeroid conditions. An *in-frame* deletion of Serine 605 (p.Ser605del) within the*POLD1* gene accounts for 78% of the patients [5]. The *POLD1* gene encodes the catalytic subunit of Polδ, which possesses both polymerase and exonuclease activities. The Ser605 deletion occurs within a highly conserved region of the polymerase active domain, which is crucial for the incorporation of dNTPs during primer extension and for phosphodiester bond formation. In vitro functional studies have shown that this mutation abolishes polymerase activity and partially impairs exonuclease function, causing DNA synthesis failure, stalled replication forks, double-stranded DNA breaks, and genomic instability—mechanisms implicated in cellular aging and death [5–7].

Polδ comprises the catalytic subunit POLD1 and three accessory subunits: POLD2, POLD3, and POLD4. The POLD1 protein contains a nuclear localization signal, an exonuclease domain, a polymerase active site, and a ZnF domain [8–10]. Polδ plays a central role in eukaryotic DNA replication, extending primers on the lagging strand with high accuracy, and it is also involved in several DNA repair pathways [11–13].

The functional role of the *in-frame* deletion and the pathological cellular phenotype has already been characterized in our previous works [13–15], highlighting a typical senescence framework that has been reconfirmed by us recently in mesenchymal stem cells obtained from the same patients [15, 16].

Considering the marked phenotypic variability and genomic instability of MDPL, RNA sequencing (RNA-seq) offers unparalleled resolution for uncovering dysregulated pathways and molecular signatures defining the disease.

In this study, RNA-seq analysis was performed to identify universally expressed transcripts across MDPL and WT HDF samples under basal conditions and after 1 Gy X-irradiation for exacerbating pathological processes and molecular signatures associated with DNA repair defects.

Our results identified a pool of genes significantly differentially expressed between MDPL HDFs and WT. This finding revealed a strong contribution of those genes involved in ECM structure and composition and, most interestingly, in adipose tissue differentiation. After DNA damage induction, a downregulation of genes involved in DNA replication checkpoint, cell cycle regulation, and nucleic acid metabolism was observed.

By characterizing these shared transcriptomic features, our work seeks to expand our understanding of MDPL pathogenesis and unveil regulatory mechanisms at the molecular and cellular levels, paving the way for future therapeutic interventions.

## Materials and methods

### Cell cultures

Two MDPL patients of different ages were recruited: POLD11 (21 years) and POLD12 (14 years), along with two age-matched healthy controls (WT-CCM00334 and CX14). After collecting the informed consent, skin punch biopsies (4 mm, Visipunch) were obtained following standard procedures. HDFs were obtained as previously reported [13].

For irradiation experiments, HDFs were exposed to 1 Gy of ionizing X-rays once they reached appropriate confluence and were collected 24 h post-treatment (+ 24 h) under standard culture conditions.

### RNA sequencing

After assessing RNA quality control (RNA 6000 Nano Kit on a Bioanalyzer, Agilent Technologies, USA), samples were quantified using the Qubit RNA BR Assay Kit (Thermo Fisher Scientific, USA) and Illumina RNA-seq libraries were generated using the TruSeq stranded mRNA ligation kit (Illumina), according to the manufacturer’s instructions. The quality and size of RNA-seq libraries were assessed by capillary electrophoretic analysis with the Agilent 4150 Tape station (Agilent Technologies, USA). Libraries were quantified by real-time PCR against a standard curve with the KAPA Library Quantification Kit (KapaBiosystems, Wilmington, MA, USA). Libraries were pooled at equimolar concentration and sequenced with Illumina technology, generating on average 22 million fragments in 150PE mode on a Novaseq6000 sequencer.

#### Gene expression analysis

Total RNA was extracted from cells with TRIzol (Ambion, Foster City, CA, USA), according to the manufacturer’s instructions, followed by reverse transcription (RT) of 1 µg of RNA using the Life Technologies Corporation’s High-Capacity cDNA Archive kit (Foster City, CA, USA). Expression analysis was performed by quantitative RT-polymerase chain reaction (SYBR Green Assay, Life Technologies Corporation, Foster City, CA, USA), using GAPDH as the internal reference gene. The comparative ΔΔCt method was used to quantify relative gene expression levels.

### Differential gene expression analysis

Differential gene expression analysis was performed using the DESeq2 package in R [17], excluding genes with expression levels below 5 in fewer than half of the samples. Differentially expressed genes (DEGs) were defined as those with a *p* < 0.05 and an absolute fold change (|FC|> 1).

### Functional enrichment analysis

To explore the biological significance of the DEGs, functional enrichment analysis was carried out using ShinyGO [18]. This tool identifies enriched Gene Ontology (GO) terms and pathways associated with the upregulated and downregulated genes.

### Protein-protein interaction network and visualization

The STRING database was used to investigate potential protein-protein interactions (PPIs) among the DEGs [19]. The resulting interaction networks were visualized and further analyzed using Cytoscape [20]. For each node in the network, additional information about the molecular function associated with the corresponding gene was incorporated, enabling a comprehensive representation of the functional roles of the DEGs within the network.

### Antibody array

Highly sensitive detection of selected proteins was assessed using RayBio® L-Series Antibody Array (Ray-Biotech, Inc., Norcross, GA, USA). Briefly, cells were grown as previously described, and protein was extracted following the manufacturer’s protocol. The array map is reported in the “Results” section.

### Statistical analyses

Statistical analysis for RT-qPCR was carried out using GraphPad Prism 10 software and the SPSS program, version 25 (IBM Corp, Armonk, NY, USA). The differences between groups were tested by *t*-test and one-way ANOVA test. Values provided in figures are the means of three independent experiments ± standard error of the mean (SEM). The level of significance was established at *p < 0.05, **p < 0.01, ***p < 0.001, and ****p < 0.0001.

## Results

### RNA sequencing of MDPL and WT HDFs

**Fig. 1:**
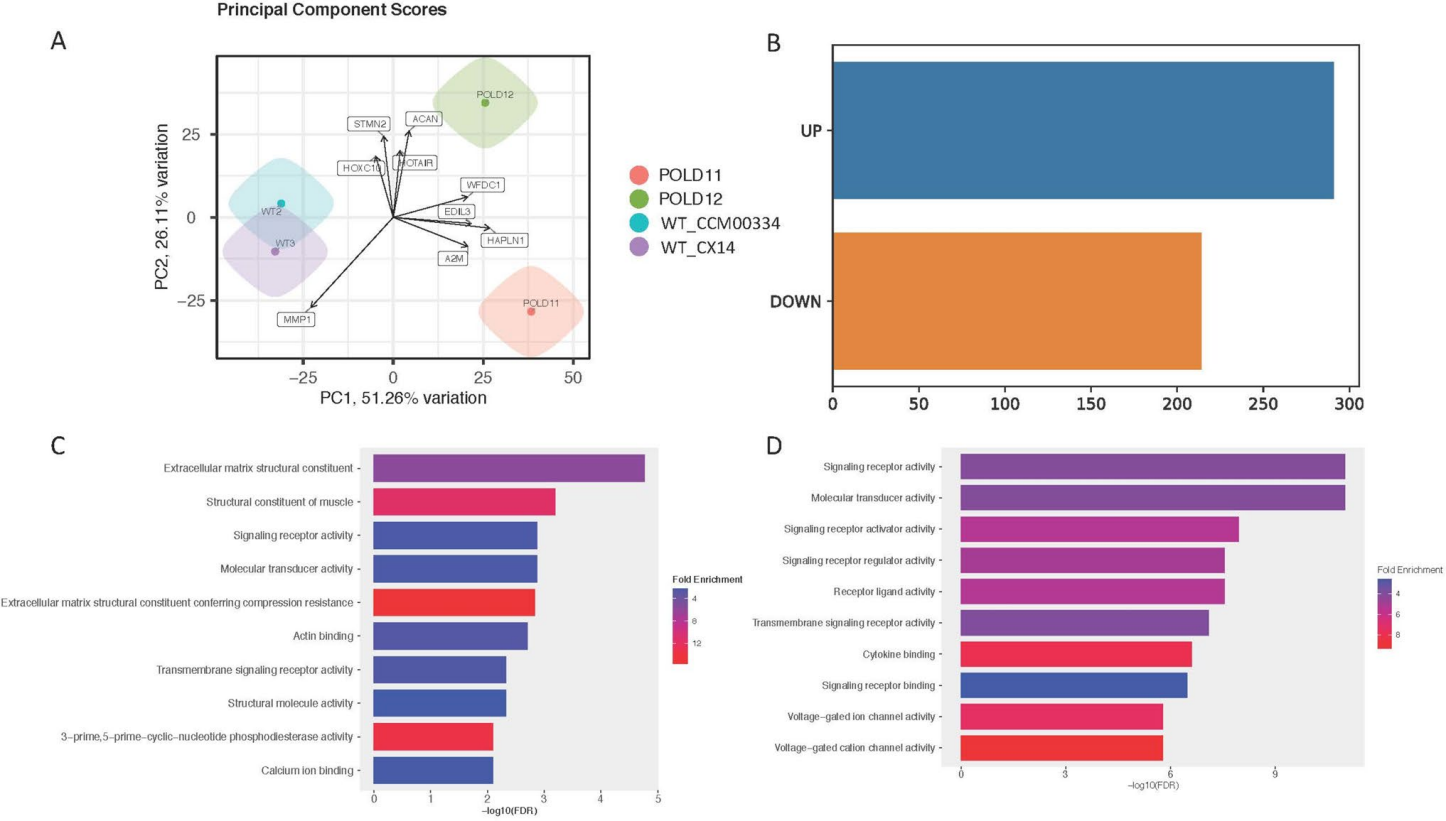
Principal component and functional enrichment analyses in MDPL and WT HDFs. **A** Principal component analysis (PCA) illustrating the distinct clustering of MDPL HDFs compared to WT ones, based on their transcriptomic profiles. **B** Number of differentially expressed genes (|FC|> 1, *p* < 0.05) between MDPL and WT HDFs. **C** Functional enrichment analysis of pathways associated with upregulated genes in MDPL patients. **D** Functional enrichment analysis of pathways associated with downregulated genes in MDPL patients

**Fig. 2:**
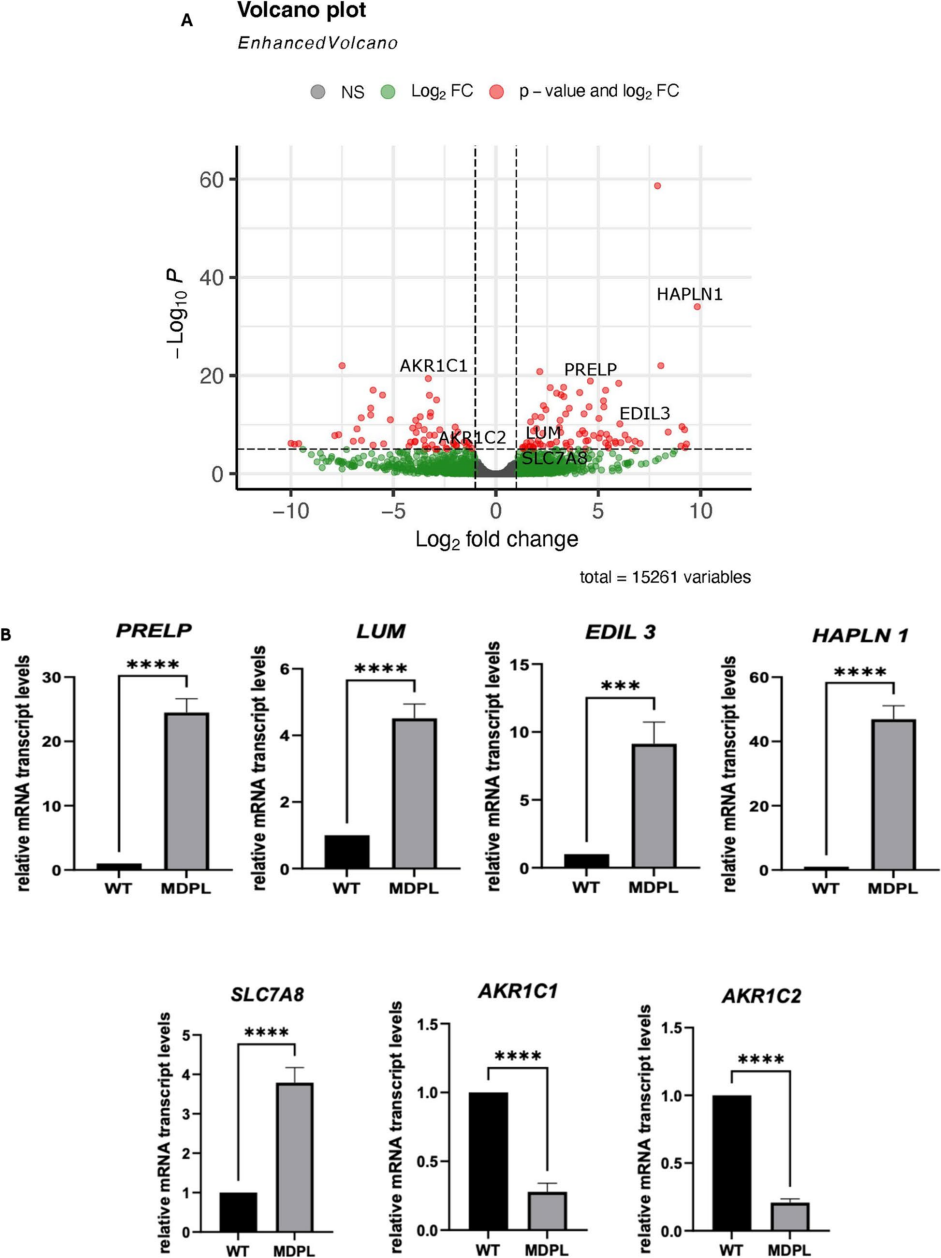
Volcano plot and RT-qPCR in MDPL compared to WT HDFs. **A** Volcano plot displaying the differential gene expression in MDPL compared to WT cells. Genes with significant differential expression (|FC|> 1, *p* < 0.05) are highlighted in red, indicating the most prominent changes in expression. **B** Quantitative real-time PCR of *PRELP*,* LUM*,* EDIL3*, *HAPLN1*, *SLC7A8*, and *AKR1C1/2* transcripts using WT as unit. GAPDH was used as the reference gene. Data are representative of three independent experiments and reported as mean ± SEM. Mean values were compared using the two-tailed Student *t*-test, for independent samples (***p < 0.001, ****p < 0.0001)

A global analysis has revealed significant changes in gene expression profiles among MDPL HDFs with age-matched healthy controls (WT HDFs), being clearly distinct (Fig. 1A). Comparative expression analysis has identified approximately 500 genes significantly differentially expressed between MDPL and WT ones (*p*_adj < 0.05): specifically, 214 genes were downregulated, and 291 genes were upregulated (Fig. 1B) (Supplementary Table 1). To gain a broader understanding of the molecular functions associated with the differentially expressed genes, we performed a functional enrichment analysis. This analysis revealed that the genes upregulated in MDPL patients are primarily involved in ECM composition, structural molecule activity, and calcium ion binding (Fig. 1C). In contrast, the downregulated genes are associated especially with molecular transducer activity, signaling receptor activity, and cytokine binding (Fig. 1D). Genes involved in the ECM, molecular transducer activity, and signaling receptor activity drew our interest due to their well-established roles in aging regulation [21]. Among them, the upregulated genes include *PRELP* (Prolargin),* LUM* (Lumican),* HAPLN1* (Hyaluronan and Proteoglycan Link protein 1), *EDIL3* (EGF-like repeat and discoidin I-like domain-containing protein 3), and *SLC7A8* (Solute Carrier Family 7 Member 8), while *AKR1C1* and *AKR1C2 (*Aldo-Keto Reductase Family 1 Member C1-2) resulted to be downregulated, as shown in the volcano plot (Fig. 2A). These findings were successively confirmed in a significant manner (***p < 0.001, ****p < 0.0001) by RT-qPCR (Fig. 2B), especially for *PRELP* and *HAPLN1* markers (50 and 24.5 times, respectively) (Fig. 2B).

*PRELP*, *LUM*, *HAPLN1*, and *EDIL3* genes encode proteins involved in forming and stabilizing ECM, showing also a significant association with bone homeostasis, skeletal system development, and muscle health [22–24]*.* Moreover, according to the literature, the expression of *PRELP* coincides with the appearance of a premature-aging disease symptoms, called HGPS (Hutchinson-Gilford Progeria Syndrome) [25]. *LUM* encodes a member of the small leucine-rich proteoglycan (SLRP) family, while *EDIL3* encodes an integrin ligand [22, 24]. *PRELP* encodes a heparin/heparan sulfate–binding protein with high affinity to collagens I and II, functioning as a linker with the extracellular matrix [26]. It is expressed in various tissues, including bone tissue, skin, and basement membranes [27–30]*. HAPLN1* specifies a very important component of the ECM, responsible for stabilizing its macromolecular structure, maintaining the binding activity of the hyaluronic acid (HA) and proteoglycan. Interestingly, *HAPLN1* expression is elevated in certain musculoskeletal diseases, such as rheumatoid arthritis (RA) [23]. Finally, *SLC7A8* is three times upregulated in MDPL patients (Fig. 2B) and encodes a large neutral amino acid transporter small subunit 2 (LAT2), responsible for the regulation of the intracellular amino acid pools. *SLC7A8* plays a role in age-related hearing loss [31] and regulates adipose tissue biology and lipid accumulation [32], both aspects closely related to MDPL syndrome.

Regarding those genes downregulated in MDPL patients, they are associated with molecular functions, including molecular transducer and signalling receptor activity. *AKR1C1* and *AKR1C2* (Fig. 2B) are strongly associated with differentiation and adipose tissue distribution [33, 34].

Thus, also in this case, data obtained from our analysis could be a starting point to better understand the pathogenetic mechanism associated with adipose tissue anomalies in MDPL patients.

### RNA sequencing analysis of MDPL and WT HDFs after 1 Gy X-irradiation

**Fig. 3:**
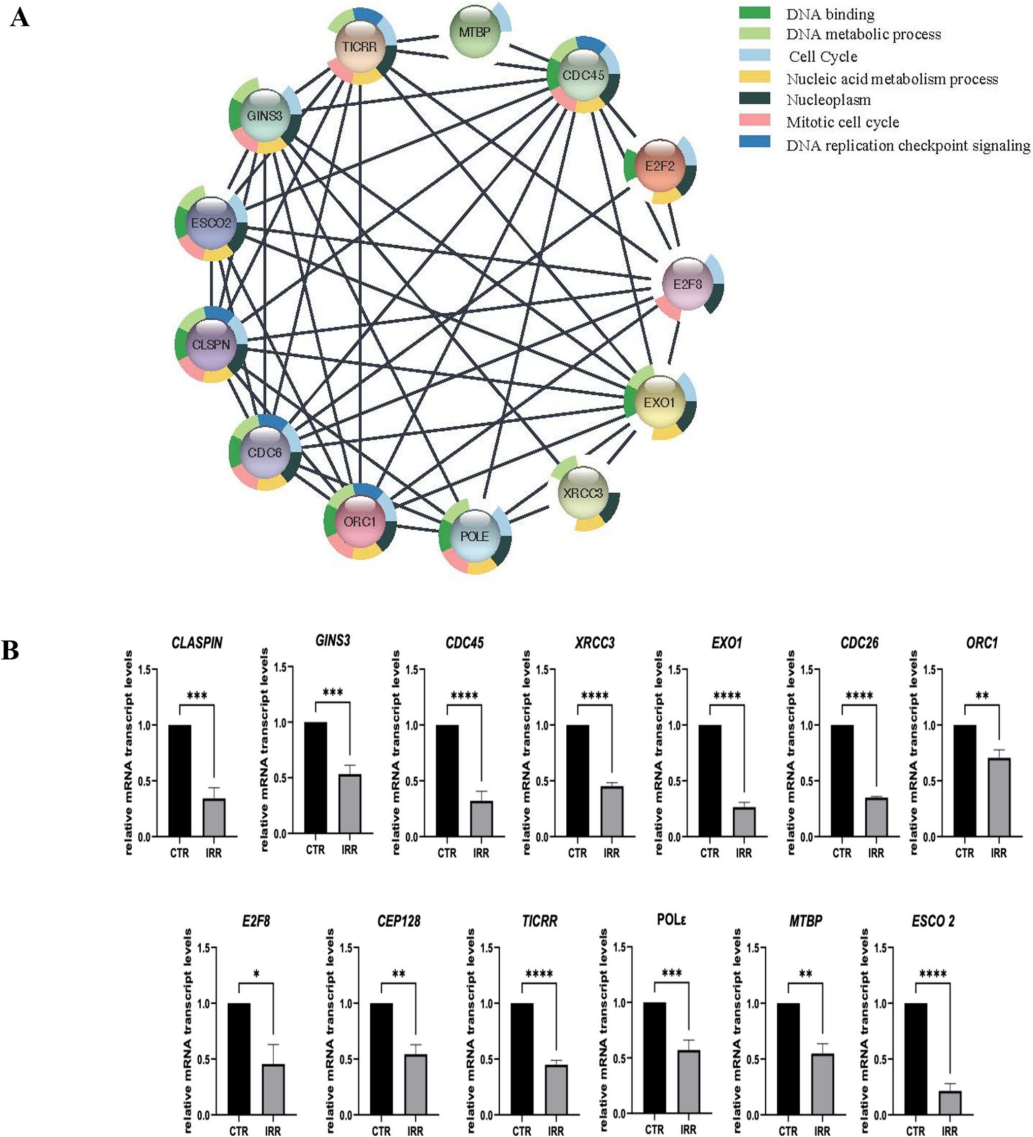
STRING and RT-qPCR analyses in irradiated HDFs MDPL compared to non-irradiated ones. **A** Network of interactions (edges) identified via STRING analysis between the downregulated genes in irradiated HDF MDPL compared to non-irradiated ones. The network highlights key gene interactions involved in biological processes such as DNA replication, cell cycle regulation, and DNA repair mechanisms. **B** RT-qPCR in irradiated MDPL HDFs vs MDPL not irradiated ones of genes involved in DNA replication checkpoint, cell cycle regulation, nucleic acid metabolism (e.g., *CLSPN*,* GINS3*,* CDC45*,* XRCC3*,* EXO1*,* CDC6*,* ORC1*,* E2F8*,* CEP128*, *TICRR*,* POL*Ɛ, *MTBP*,* ESCO2*), and DNA binding using WT as unit. GAPDH was used as reference gene. Data are representative of three independent experiments and reported as mean ± SEM. Mean values were compared using the two-tailed Student *t*-test, for independent samples (*p < 0.05, **p < 0.01, ***p < 0.001, ****p < 0.0001)

Considering that POLD1 is a central mediator of DNA replication and repair, we then exposed MDPL HDFs to 1 Gy of X-irradiation to determine the impact of Ser605 deletion on the response to cellular damage and repair. Thus, we compared the expression profiles among MDPL HDFs irradiated and not, by using RNA sequencing to identify and confirm those differentially expressed markers involved in DNA repair pathways (Supplementary Table 2). The analysis revealed approximately 50 downregulated genes in irradiated HDFs samples (log2FC ≤ −1), such as CLSPN (Claspin), XRCC3 (X-Ray Repair Cross Complementing 3), E2F8 (E2F Transcription Factor 8), E2F2 (E2F Transcription Factor 2), CDC45 (Cell Division Cycle 45), GINS3 (GINS Complex Subunit 3), ESCO2 (Establishment Of Sister Chromatid Cohesion N-Acetyltransferase 2), CDC6 (Cell Division Cycle 6), ORC1 (Origin Recognition Complex Subunit 1), EXO1 (Exonuclease 1), TICRR (TOPBP1 Interacting Checkpoint And Replication Regulator), MTBP (MDM2 Binding Protein), CEP128 (Centrosomal Protein 128), and POLE (DNA Polymerase Epsilon, Catalytic Subunit) (Fig. 3A). These genes are enriched in biological processes such as DNA replication checkpoint, cell cycle regulation, nucleic acid metabolism, and DNA binding.

The gene expression of these markers was confirmed by RT-qPCR, corroborating the downregulation of most of these genes (*p < 0.05, **p < 0.01, ***p < 0.001, ****p < 0.0001) (Fig. 3B).

The significant reduction in the expression of these key markers suggests a substantial cellular defect in modulating their responses to DNA damage and triggering repair mechanisms in MDPL syndrome.

**Fig. 4:**
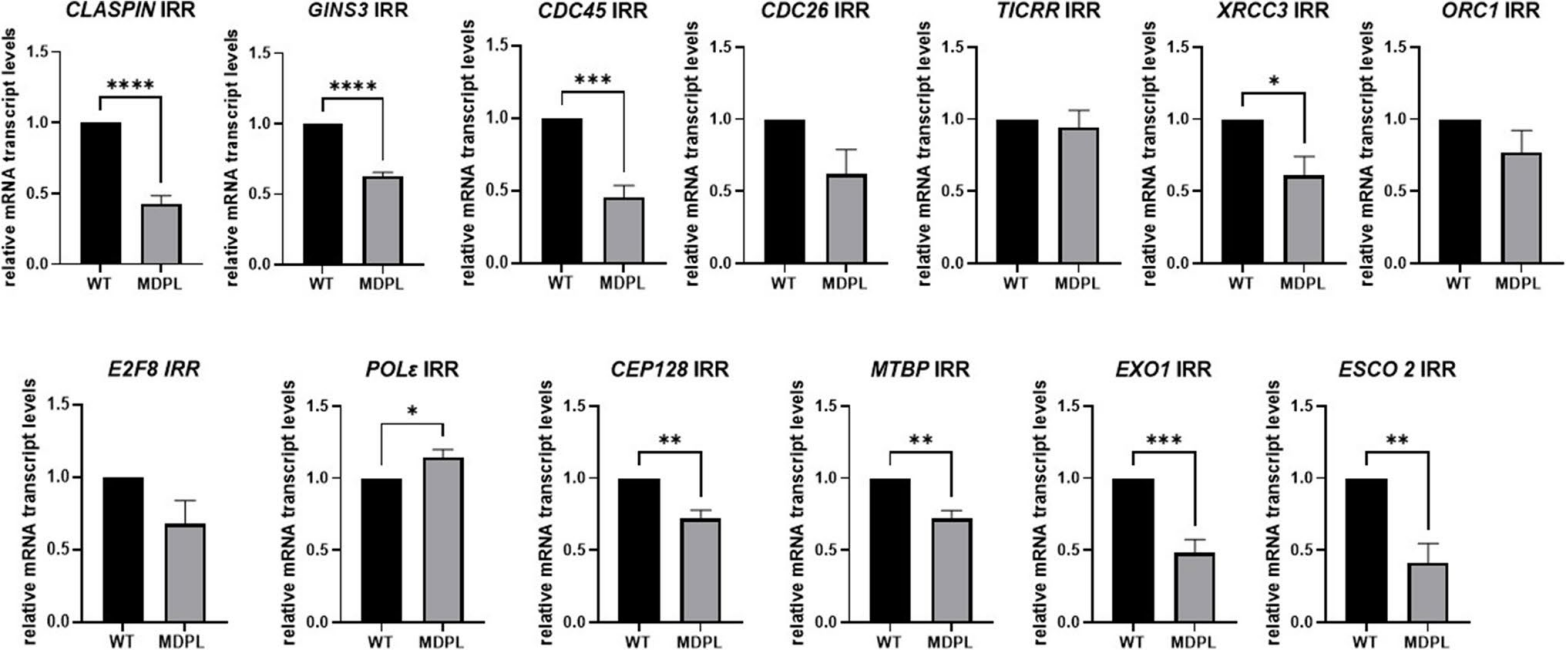
RT qPCR in irradiated MDPL HDFs vs irradiated WT HDFs. RT-qPCR in irradiated MDPL HDFs vs irradiated WTof *CLSPN*,* GINS3*,* CDC45*,* XRCC3*,* EXO1*,* CDC6*,* ORC1*,* E2F8*,* CEP128*, *TICRR*,* POL*Ɛ, *MTBP*, and *ESCO2* markers was quantified using WT as unit. GAPDH was used as reference gene. Data are representative of three independent experiments and reported as mean ± SEM. Mean values were compared using the two-tailed Student *t*-test, for independent samples (*p < 0.05, **p < 0.01, ***p < 0.001, ****p < 0.0001)

To evaluate whether the involvement of genes responsible for DNA replication and repair was only specifically linked to exposure to 1 Gy irradiation, regardless of cell genotype, we also compared the quantitative expression of irradiated WT HDF samples versus non-irradiated ones. From this comparison, no genes were found to be significantly differentially expressed (|FC|>  1 and *p* < 0.05), highlighting the specificity link to the pathogenetic genotype (data not shown). To strengthen our conclusions, we finally compared irradiated MDPL HDFs with irradiated WT ones, as shown in Fig. 4. In this way, by RT-qPCR, we further assessed the downregulation of these genes in irradiated MDPL HDFs, corroborating the specificity in dysregulation of these markers, which results in being strictly linked to the genotype and not to the irradiation phenomenon.

### Protein analysis by highly sensitive antibody array

Transcriptomic analysis identified a network of genes involved in DNA replication, cell cycle checkpoint regulation, and DNA damage repair, suggesting a key role of these processes in aging pathology.

**Fig. 5:**
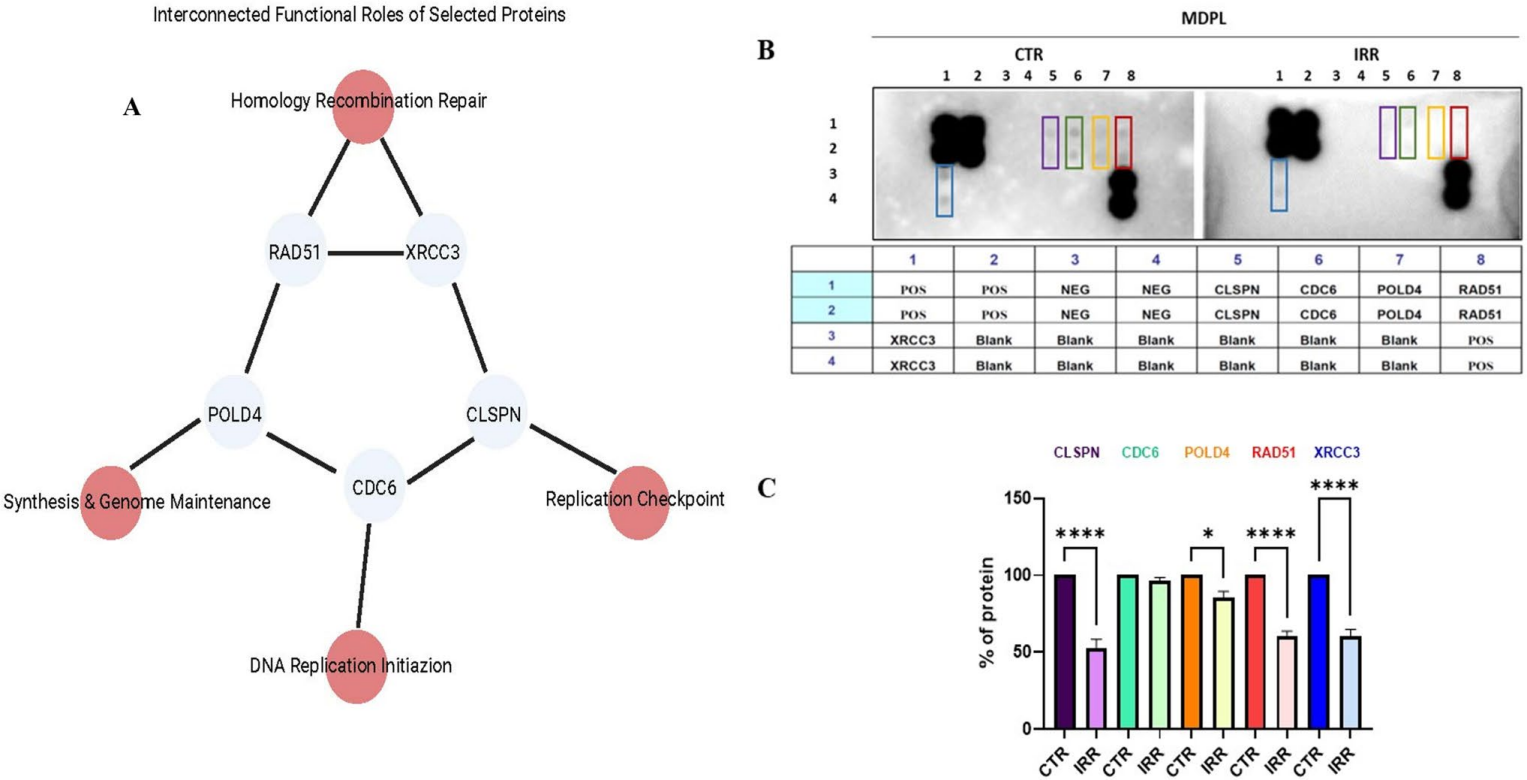
Network representation of the selected proteins and antibody array analysis. **A** Network representation of the selected proteins (CDC6, CLSPN, XRCC3, RAD51, and POLD4, in blue) and their functional roles (in red) in genomic stability. CDC6 regulates DNA replication initiation; CLSPN mediates the replication checkpoint; XRCC3 and RAD51 facilitate homologous recombination repair, and POLD4 contributes to DNA synthesis and genome maintenance. The interconnections highlight their coordinated roles in replication and DNA damage response. **B** Antibody array map and target list of a representative blot assay reporting all protein markers evaluated. **C** Bar graph illustrating the densitometric analysis of the relevant proteins modulated by 1 Gy X-irradiation in MDPL HDFs. Data are reported as mean ± SEM (*n* = 4) (*p < 0.05, ****p < 0.0001)

To investigate these findings at the functional level, we selected four proteins—CDC6, CLSPN, XRCC3, and RAD51—for their validation, given their roles in interconnected pathways ensuring genomic stability. Specifically, CDC6 regulates DNA replication initiation, CLSPN (Claspin) mediates the replication checkpoint, and XRCC3 and RAD51 facilitate homologous recombination repair of replication-associated DNA damage. This selection enables us to link transcriptional alterations to functional protein-level changes, providing insights into replication stress and DNA repair mechanisms. We also deepened the protein expression of *POLD4*because its role has recently been described as crucial for genome maintenance [35] (Fig. 5A). Except for CDC6, all proteins resulted, as expected, in being downregulated in irradiated MDPL HDFs, with respect to the corresponding controls (CTRs) (*p < 0.05, ****p < 0.0001) (Fig. 5B, C). This data further underlies the pathological role played by mutated Polδ polymerase in the cellular DNA repair mechanism.

## Discussion

Transcriptome profiling represents an opportunity for biomarker identification, and the comparison of transcriptomic readouts within or between individual samples allows the identification of outlier molecular signatures.

By using an integrated transcriptomics approach, we have created a comprehensive map of complex gene expression networks in MDPL, which sheds light on intricate interactions within cellular signalling pathways.

The precise pathogenic mechanisms underlying MDPL syndrome remain poorly understood, and, in this context, our study performed on MDPL HDFs is the first attempt to reveal their characteristic molecular signatures and disease mechanisms.

MDPL patients commonly present with skeletal abnormalities such as sclerodermatous skin, mandibular hypoplasia, sensorineural hearing loss, and lipodystrophy [13, 36]. Clinical features progressively emerge, particularly the gradual loss and redistribution of subcutaneous fat in late childhood, around the first decade of life [37, 38]. Dysmorphic features include mandibular hypoplasia, prominent eyes, beaked nose, narrow mouth with dental crowding, progeroid appearance, and thin skin—sometimes with telangiectasia. Additional findings include joint contractures, early-onset osteoporosis, thoracic kyphosis, and scoliosis. Hearing loss, typically developing in the first or second decade, is a hallmark of MDPL, though exceptions have been reported [5–9, 36, 38–40].

In this study, RNA sequencing analysis was performed to identify universally expressed transcripts in HDFs obtained from MDPL patients and age-matched healthy subjects. The analysis was done under basal conditions and after one Gy X-irradiation, aimed at exacerbating pathological processes and highlighting molecular signatures associated with DNA repair defects.

At the basal level, we observed in MDPL patients differentially expressed genes encoding proteins related to extracellular matrix constituents, structural molecule activity, and calcium ion binding. Other pathways involved are molecular transducer, signalling receptor activities, and cytokine binding. Specifically, among those genes, we found *PRELP*, which encodes a small leucine-rich proteoglycan (SLRP) protein and whose role is to bind type I collagen to basement membranes and type II collagen to cartilage. Also, *LUM* belongs to the SLRP protein family, while *HAPLN1* is responsible for stabilizing the macromolecular structure of the ECM. Finally, *EDIL3*, an integrin ligand involved in the ECM structure, aims at maintaining the structure and the function of the extracellular matrix, as well as enabling cell adhesion.

Interestingly, recent reviews have described among progeroid diseases those associated with different molecular defects, including connective tissue alterations [41]. During aging, typical changes in the structure of the dermis and a loss of ECM integrity are observed. Particularly, recent studies have demonstrated changes in ECM composition, leading to a stiffer and mechanistically weaker ECM in old age [1, 42].

*PRELP*, *LUM*, *HAPLN1*, and *EDIL3* transcripts result upregulated in MDPL HDFs and show a significant association with bone homeostasis, skeletal system development, and muscle health [22–24]. Also,*PRELP* expression has been reported with the appearance of symptoms described in another well-known premature-aging disease, such as HGPS [25].

Finally, we investigated *SLC7A8*, a neutral amino acid transmembrane transporter involved in age-related hearing loss [31]. Thus, we can speculate that its higher expression in MDPL patients (higher than three times: ****p < 0.0001) could be linked to the deafness phenotype observed in some of them.

*SCL7A8* is also known to regulate adipose tissue biology and lipid accumulation [32], one of the primary target tissues involved in MDPL syndrome.

Among those genes downregulated at the basal level, AKR1C1 and AKR1C2 are members of the aldoketo reductase (AKRs) family and are downregulated in MDPL cells (****p < 0.0001). AKR1C1 and AKR1C2 are strongly associated with differentiation and adipose tissue distribution [33, 34], an aspect we are currently deepening. These data shed light on an initial framework that aids in comprehending the disease-causing processes linked to abnormalities in adipose tissue and in the extracellular matrix among MDPL patients.

In order to stress the cellular and DNA repair system that is notoriously defective in MDPL cells, we irradiated them as already established in our previous studies [13]. Expression analysis revealed most of the key elements involved in biological processes, such as DNA replication checkpoint, cell cycle regulation, nucleic acid metabolism, and DNA binding. All of them are downregulated in irradiated MDPL HDFs, emphasizing the functional strength of the machine placed at the shelter of the damage.

Polδ is a crucial enzyme involved in the synthesis of newly formed DNA strands in the eukaryotic genome. It is made up of four subunits: the catalytic POLD1 and non-catalytic subunits POLD2, POLD3, and POLD4. The loss of POLD4 in human lung cancer cells leads to genomic instability, suggesting a novel function for POLD4 in controlling Polδ’s response to DNA damage [43]. Its underexpression in MDPL irradiated might hypothesize the dynamic transitions of Polδ for crucial genome maintenance. Nevertheless, the exact role of POLD4 remains to be fully elucidated.

Transcriptome analysis has identified a network of genes involved in DNA replication, cell cycle checkpoint regulation, and DNA damage repair, suggesting that these processes play a crucial role in MDPL pathology. To further explore these findings at the functional level, we selected and deepened four pivotal proteins—CDC6, CLSPN, XRCC3, and RAD51—for protein validation, due to their involvement in interconnected pathways that maintain genomic stability.

CLSPN was found downregulated at both transcript and protein levels in MDPL patients. As one of five genome stability factors, it works with CDC6—activated under moderate replication stress and fork-associated factors like RAD51—to support replication fork progression by coordinating helicase activity and DNA polymerization. Smits et al. described CLSPN’s roles in DNA replication, including direct DNA binding and interaction with replisome components [44]. The marked reduction of CLSPN protein post-irradiation in our patients may impair fork progression, leading to increased fork collapse due to polymerase-helicase uncoupling, excess single-stranded DNA exposure, and/or reduced polymerization [45]. Additionally, XRCC3 modulates fork progression in damaged chromosomes through its role in homologous recombination with RAD51. As a RAD51-interacting protein with limited sequence similarity, XRCC3 is essential for homology-directed DNA repair, a key process in maintaining genomic integrity in vertebrates.

## Conclusion

To date, no clear explanation has been provided linking gene activities to the clinical features of MDPL patients. While our findings provide novel insights into the molecular landscape of MDPL, the extremely rare nature of the genetic disorder restricts our ability to establish strong genotype-phenotype correlations. Future studies on a larger patient cohort and additional affected tissues will be essential to validate these findings and better understand the link between lipodystrophy and cellular senescence in MDPL.

Also, knockdown or overexpression experiments will be necessary to clarify the role played by these molecular pathways in MDPL pathogenesis and in the therapeutic management. Targeting these dysregulated pathways could represent a promising direction for future research.

## Supplementary Information

Below is the link to the electronic supplementary material.

ESM 1 (XLSX 3.23 MB)

ESM 2 (XLSX 3.23 MB)

## Data Availability

The original contributions presented in the study are included in the article, and further inquiries can be directed to the corresponding authors.
